# Diethylstilbestrol in the Treatment of Castration-resistant Prostate Cancer: A Lower-middle-income Country Experience

**DOI:** 10.7759/cureus.4470

**Published:** 2019-04-16

**Authors:** Azfar Ali, Muhammad Arshad Irshad Khalil, Nouman Khan, Muhammad Abu Bakar, Awais Amjad, Irfan Ahmed, Khurram Mir

**Affiliations:** 1 Surgical Oncology, Shaukat Khanum Memorial Cancer Hospital and Research Center, Lahore, PAK; 2 Biostatistics and Epidemiology, Shaukat Khanum Memorial Cancer Hospital and Research Center, Lahore, PAK; 3 Surgery, Shaukat Khanum Memorial Cancer Hospital and Research Center, Lahore, PAK; 4 Urology, Shaukat Khanum Memorial Cancer Hospital and Research Center, Lahore, PAK

**Keywords:** castrate resistant prostate cancer, diethylstilbestrol

## Abstract

Introduction: Prostate cancer is the second most common cancer and the fifth leading cause of death worldwide. Its metastatic stage is associated with considerable morbidity and may lead to death. In Pakistan, given the high levels of economic constraint, patients with castration-resistant metastatic prostate cancer can be treated with cost-effective medications like diethylstilbestrol (DES).

Objectives: The goal of this study was to assess the efficacy and adverse effects of DES when used in patients with castration-resistant prostate cancer (CRPC).

Materials and methods: From January 2011 to December 2016, all medical records of patients with a diagnosis of prostate cancer resistant to the effects of castration presenting at Shaukat Khanum Cancer Hospital and Research Centre, Lahore, were reviewed. All patients were treated with DES (2.5 mg) initially, but the dose was increased for some patients to 5 mg in combination with aspirin (75 mg). The patients were followed clinically with prostate-specific antigen (PSA) value assessment. The PSA response to treatment, time to disease progression, and adverse events were recorded and analyzed using IBM SPSS Statistics for Windows, Version 20.0 (IBM Corp., Armonk, NY).

Results: A total of 91 patients were included in the study, and the mean patient age was 66 ± 8 years. The median baseline PSA was 150 ng/mL (range: 56-626 ng/mL), and the median Gleason’s score was eight. A total of 90.1% of patients had metastatic disease at the time of diagnosis. Hormonal ablation was provided with bilateral orchiectomy for 71 patients (78.0%), and luteinizing hormone-releasing hormone (LHRH) analog was provided for 20 patients (22.0%). With this treatment, the median time to PSA progression was 597 days. After DES treatment was started, 78 patients (87.7%) showed a PSA response, and median time to progression was 212 days. In 24 patients (26.4%), the PSA response was maintained for more than a year. The PSA response was quantified as a good response (i.e., ≥50% PSA drop) or as a partial response (i.e., <50% PSA drop). The good PSA response was observed in 56 patients (61.5%) with a median time to progression of 273 days, and 22 patients (24.2%) had a partial response maintained for 109 days. Thirteen patients (14.3%) did not respond to DES treatment. The median percent change in PSA was -55.52% (range: -99.9 to +422). Thromboembolic complication was observed in eight patients (8.7%) patients while two patients suffered from liver toxicity.

Conclusion: DES is an effective, economical, and relatively safe drug in patients with CRPC.

## Introduction

Prostate cancer is a hormone-responsive disease. Androgen ablation is used as a primary treatment in symptomatic metastatic disease and as an adjuvant to radiation treatment in localized moderate- to high-risk cases [1]. Androgen deprivation therapy is achieved either by luteinizing hormone-releasing hormone (LHRH) analogs or bilateral orchiectomy. Approximately 90% of patients respond well to the first line of hormonal treatment for a median time of 18-24 months [2]. In due course, however, most of these patients develop progressive disease even after the castrate levels of testosterone are achieved. This becomes evident by the progressive rise in serum prostate-specific antigen (PSA) levels leading to increased numbers of bony metastatic lesions seen via bone scan. At this time, the patient develops castration-resistant prostate cancer (CRPC), and few treatment options are available. CRPC can be treated with systemic chemotherapy with compounds such as docetaxel and cabazitaxel which carries a definite survival benefit [3-4]. However, chemotherapy is accompanied by its inherent complications, and patients’ tolerance becomes the limiting factor.

Further hormonal manipulation in CRPC patients can be achieved via agents including estrogens, abiraterone, and enzalutamide [5]. An overall survival benefit is achieved with the latter two agents, but still, 10%-20% of patients are refractory to this form of treatment [6]. Additionally, a considerable cost of these medications has limited their wider use in lower-middle-income countries like Pakistan.

Diethylstilbestrol (DES) is a synthetic estrogen that has shown efficacy in the treatment of prostate cancer dating back to the 1940s [7]. Orchiectomy and DES were used as first-line treatments for the next two decades until studies by the Veterans Administration Co-operative Urological Research Group (VCURG), reported significant thromboembolic events caused by DES at doses of 5 mg daily [8-9]. The development and use of the LHRH analogs further decreased the use of DES in the treatment of prostate cancer because LHRH offered similar efficacy to that of orchiectomy with less physical and psychological trauma, and LHRH analogs did not cause increased thromboembolic complications as was seen with DES. Subsequently, the VCURG II study and some other smaller studies suggest that low doses of DES showed similar clinical efficacy as the higher dose with fewer cardiovascular side effects [10-12]. DES with thromboembolic prophylaxis has proven to be a less expensive alternative to the new costly hormonal agents, forcing clinicians to reconsider DES use in the management of CRPC. With its low cost and proven efficacy, we used it as a second-line treatment in combination with anticoagulant agents in patients with prostate cancer who developed resistance to complete androgen blockade (CAB).

## Materials and methods

From January 2011 to December 2016, all medical records of patients with a diagnosis of prostate cancer presenting at Shaukat Khanum Memorial Cancer Hospital and Research Centre, Lahore, were retrospectively reviewed. The patients with metastatic prostate cancer receiving castration either surgically by bilateral orchiectomy or medically by LHRH analogs were identified. This group of patients was followed clinically and with PSA levels at three monthly intervals, and those patients developing biochemical failure (i.e., rising PSA levels) were offered bicalutamide in a dose of 50-100 mg to achieve CAB. The cohort of patients showing a further rise in PSA after CAB were diagnosed with CRPC and selected for our study. This was further confirmed by serum testosterone levels below the castrate level of 50 ng/dL. These patients were treated with 2.5 mg DES initially, with some progressing to 5 mg once daily. All patients were also prescribed aspirin (75 mg) once daily for anticoagulation. Patients with a lack of complete follow-up information were excluded from the study. Patients who were on LHRH analog therapy remained on this treatment to maintain the castrate level of testosterone. These patients were further followed-up at three monthly intervals (or sooner if clinically indicated) to assess PSA levels. Radiological investigations like CT scan and bone scan were performed when indicated. The PSA response to treatment, time to disease progression, and adverse events were recorded and analyzed by IBM SPSS Statistics for Windows, Version 20.0. (IBM Corp., Armonk, NY).

Prostate-specific antigen before the start of DES therapy was taken as a baseline, and the lowest PSA value achieved after initiation of DES was labeled as the nadir value. The PSA response was quantified as good when a ≥50% PSA decrease from baseline was achieved and considered as partial when the PSA response was <50% from baseline. PSA progression was defined as >50% PSA increase from the nadir values.

## Results

A total of 91 patients were included in the study. The mean age was 66 ± 8 years, and the median baseline PSA at time of diagnosis was 150 ng/mL (range: 56-626 ng/mL). Most of the patients were diagnosed with prostate cancer via transurethral ultrasound-guided biopsy and transurethral resection of the prostate (i.e., 36 [39.6%] in each group). The median Gleason’s score was eight. Eighty-two patients (90%) had metastatic disease at the time of diagnosis. Hormonal ablation was achieved by LHRH analog in 20 patients (22.0%) and bilateral orchiectomy in 64 patients (70.3%). Seven of the patients (7.7%), were initially provided LHRH analog and later underwent orchiectomy. The median decrease in PSA on androgen deprivation therapy (ADT) was -97.91% (range: -99.9%-30.88%). The time needed for PSA progression was from 6.34 to 68.94 months with a median of 19.62 months. The patients’ characteristics at the time of diagnosis are shown in Table 1.

**Table 1:** Patients’ characteristics. SD, standard deviation; TVP, transvesical prostatectomy; TURP, transurethral resection of the prostate; TRUS, transurethral ultrasound; GS, Gleason’s score; PSA, prostate-specific antigen.

Characteristic	Patient data (N = 91)
Patient age (years; mean±SD)	66±8
Mode of diagnosis	n (%)
TVP	13 (14.3%)
TURP	36 (39.6%)
TRUS Biopsy	36 (39.6%
Biopsy of distant metastases	n (%)
Bone biopsy	2 (2.1%)
Para-aortic lymph node biopsy	2 (2.1%)
Bone marrow biopsy	1 (1.1%)
Pelvic mass biopsy	1 (1.1%)
Gleason’s score distribution	n (%)
GS 6	3 (3.3%)
GS 7	31 (34.1%)
GS 8–10	55 (60.4%)
Distant metastases at diagnosis	n (%)
Yes	82 (90.1%)
No	9 (9.9%)
PSA at diagnosis, median (range)	150 (56–626)

The response to ADT is shown in Table 2.

**Table 2:** Types and response to first-line hormonal treatment. LHRH, luteinizing hormone-releasing hormone; PSA, prostate-specific antigen; PFD, progression free duration.

Primary hormonal treatment	n (%)
LHRH analog	20 (22.0%)
Bilateral orchiectomy	64 (70.3%)
LHRH followed by orchiectomy	7 (7.7%)
Change in PSA%, median (range)	-97.91 (-99.9, -30.88)
PFD months, median (range)	19.62 (6.34, 68.94)

The patients’ response to DES therapy is shown in Table 3.

**Table 3:** PSA response to DES therapy. PSA, prostate-specific antigen; DES, diethylstilbestrol; PFD, progression free duration; NA, not applicable.

Therapy	Median (range)	
PSA at the start of therapy	68.45 ng/mL (4.48-1,639 ng/mL)	
Follow-up months	13.6 (6.5-22)	
Overall PSA response to DES	n (%)	
Yes	78 (85.7%)	
No	13 (14.3%)	
Change in PSA%, median (range)	-55.52 (-99.9, +422)(	
Overall PFD, median (range)	6.98 months (1.77–34.38)	
PSA response to DES	n (%)	Median time to progression
≥50% PSA response	56 (61.5%)	8.96 months
<50% PSA response	22 (24.2%)	3.5 months
No response	13 (14.3%)	NA

**Figure 1:**
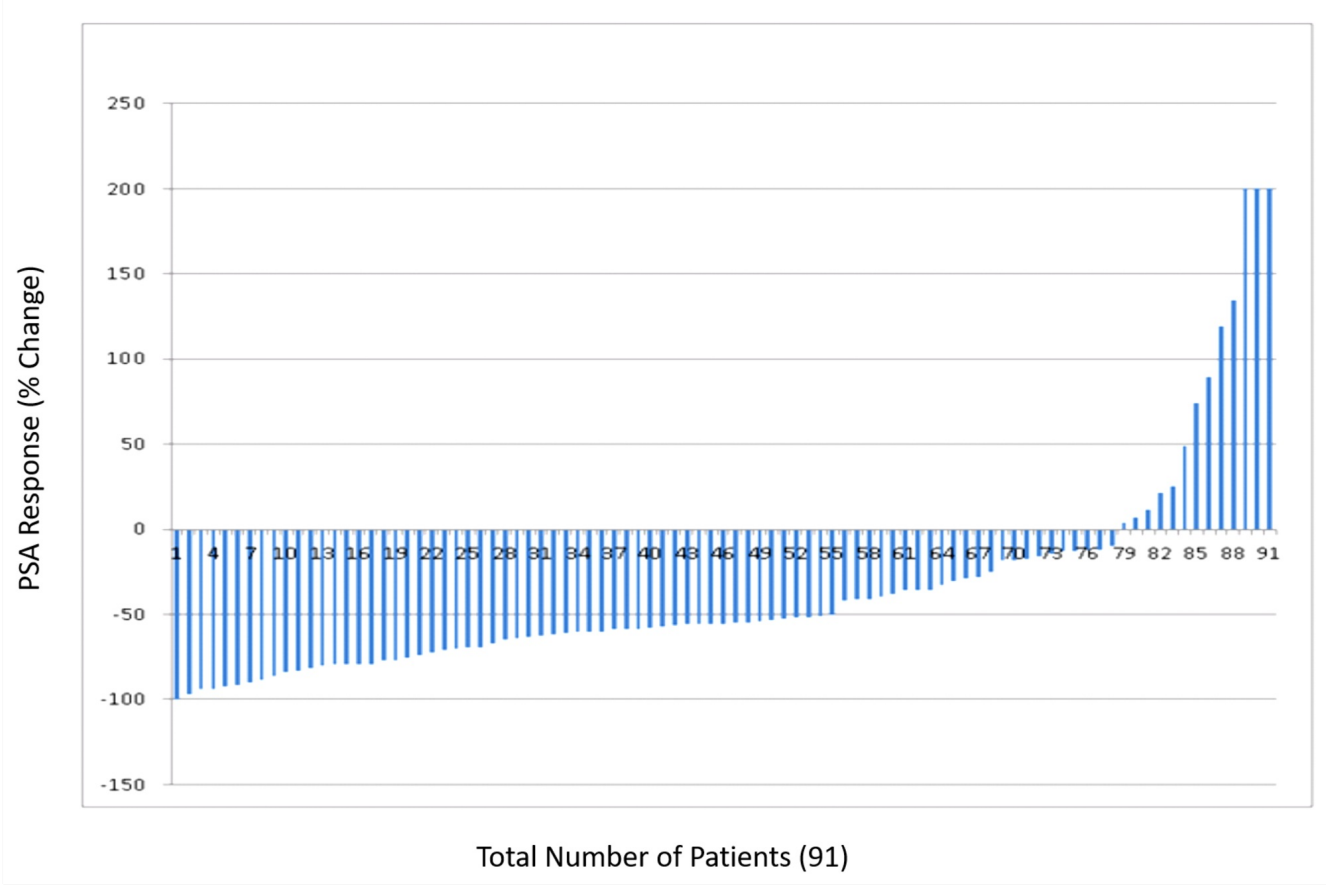
Water fall plot showing variations in PSA response to DES therapy. PSA, prostate-specific antigen; DES, diethylstilbestrol.

The variations in PSA response to DES therapy is shown in Figure 1.

Further treatments for patients in whom DES therapy failed are presented in Table 4.

**Table 4:** Further treatment after DES failure. DES, diethylstilbestrol.

Treatment	n (%)
Palliative chemotherapy	26 (28.6%)
Abiraterone	4 (4.4%)
Ketoconazole	1 (1.1%)
Palliative care	31 (34.1%)

The detail of patients suffering from complications after using DES is shown in Table 5.

**Table 5:** Adverse effects of DES therapy. DES, diethylstilbestrol.

DES adverse effects	n (%)
Deep venous thrombosis	2 (2.2%)
Myocardial infarction	4 (4.4%)
Pulmonary embolism	1 (1.1%)
Cerebrovascular accident	1 (1.1%)
Liver toxicity	2 (2.2%)
Gynecomastia	11 (12.08%)

Patients suffering from gynecomastia were managed with irradiation in nine patients; two required subcutaneous mastectomies for symptomatic relief. Two patients suffered from liver toxicity and DES had to be stopped.

## Discussion

Diethylstilbestrol is a synthetic estrogen first manufactured in 1938. Its exact mechanism of action in CRPC is not clear, but it appears to be multifactorial. DES reduces serum testosterone levels by suppressing the hypothalamic testicular axis [13]. It also induces changes in adrenal androgen, dehydroandrostenedione (DHEA), and its sulfate derivative (DHEAS). It demonstrates a direct apoptotic effect on prostate cancer cells and inhibits DNA synthesis and angiogenesis [14-15].

In our study, DES appears to have significant activity in CRPC patients as 78% of patients showed an overall PSA response to it. A PSA response of ≥50% was achieved in 56 patients (61.5%), a rate similar to a previously published series in which DES was used as second-line hormonal therapy showing a response rate of 43% and 63% of patients [16-17]. In our series, it was also observed that patients who showed a PSA response ≥50% had a longer duration of stable disease as compared to patients who had a <50% PSA response. This finding was also substantiated in studies by other authors. Shamash et al. observed that PSA decline >50% one month after commencement of DES and dexamethasone therapy was found to predict a favorable prognosis with a median time to PSA progression more than one year and median survival of more than one year [18].

The major limitation in the use of DES for patients with CRPC is its availability. The fact that this medication is manufactured by selected pharmaceutical companies in a fixed dose formulation limits the prescription options. In our series, the drug was prescribed in a dose of 2.5-5 mg only. Because DES is available in 5-mg tablets, we could halve the tablets to create the 2.5-mg dose. Even with this relatively high dose administration, the drug tolerance profile was reasonable in most of our patients. Only eight (8.7%) patients experienced thromboembolic events. In earlier studies, anticoagulants were not used routinely. Chang et al. reported a higher incidence of cardiovascular events in the DES group compared to the flutamide group (33.3% vs. 17.6%, respectively) [19]. Regular use of anticoagulants with DES significantly reduces cardiovascular complications [20]. Bosset et al. reported a 5% incidence rate of thromboembolic event in patients taking DES with anticoagulants [21]. Many studies confirmed the use of anticoagulants with DES reduces thromboembolic events, but no particular anticoagulant has been recommended. Comparing warfarin with aspirin, Oh et al. reported a 9% thromboembolic event with daily 2-mg warfarin use, which was similar to that seen with aspirin [22].

Gynecomastia, although not bothersome, was reported in 11 of our patients. This can be prevented by prophylactic irradiation to the breast at a dose of 4-6 Gy either as a single or divided doses. Established gynecomastia is difficult to treat but may be managed with external irradiation to prevent further progression or by subcutaneous mastectomy [23-24]. Liver toxicity with deranged liver function test (LFT) results was seen in two patients. On cessation of DES, the LFTs results returned to normal.

The present study has several limitations. The data were retrospectively retrieved, and the study cohort was small. Imaging assessment to measure the response or progression was not routinely performed. Despite these limitations, the study revealed a significant response to DES in patients with CRPC.

As mentioned earlier, newer agents including abiraterone and enzalutamide have been developed noting the persistent activity of the androgen receptors in CRPC. The high costs become a major limitation to their use in patients in our part of the world. Because of its lower cost and considerable efficacy, DES is an effective alternative to these newer agents, despite its known adverse effects, until the newer treatments become available at more affordable costs.

## Conclusions

Diethylstilbestrol is an effective treatment option in patients with CRPC for a modest duration and is safer when used with aspirin. It is a logical choice in lower middle-economy countries like Pakistan, where the more expensive newer hormonal therapies are financially inaccessible for a major part of the population.
